# State Tele-Buprenorphine Prescribing Policies by Medical Professional Type

**DOI:** 10.1001/jamahealthforum.2026.0420

**Published:** 2026-04-24

**Authors:** Jessica L. Sousa, Rachel K. Landis, Ben Senator, Shona Olalere, Jessica Rigsby, Lexie Minarik, Phoebe Levine, Desiree’ Anderson, Arthur Robin Williams, Laura J. Faherty

**Affiliations:** 1 RAND, Boston, Massachusetts; 2 RAND, Arlington, Virginia; 3 RAND, Santa Monica, California; 4 Ophelia Health, Nashville, Tennessee; 5 Ophelia Health, Norfolk, Virginia; 6 RAND, Pittsburgh, Pennsylvania; 7 Columbia University Medical Center, New York, New York; 8 Ophelia Health, New York, New York; 9 MaineHealth Barbara Bush Children’s Hospital, Portland, Maine; 10 Tufts University School of Medicine, Boston, Massachusetts

## Abstract

This cross-sectional study assesses variation in state policy support for fully virtual tele-buprenorphine care for Medicaid enrollees as of August 2025 through a systematic legal mapping analysis of statutes, regulations, and Medicaid policies across all 50 US states and the District of Columbia.

## Introduction

Opioid use disorder (OUD) is a leading cause of death in the US, yet many patients face barriers to accessing buprenorphine, an evidence-based treatment.^1^ Since March 2020, the temporary suspension of the Ryan Haight Act’s in-person evaluation requirement has enabled patients with OUD to receive buprenorphine via telemedicine (tele-buprenorphine) without an initial in-person visit. Studies show tele-buprenorphine improves treatment retention.^2^

Proposed rulemaking by the US Drug Enforcement Administration (DEA) would create a special registration pathway under the Ryan Haight Act^3^ to permanently allow fully virtual OUD care by authorized physicians, nurse practitioners (NPs), and physician assistants (PAs), where permitted under state law and with safeguards for identity verification and diversion monitoring.^4^

Given this federal policy context, we assessed variation in state policy support for fully virtual tele-buprenorphine care for Medicaid enrollees through a systematic legal mapping analysis of statutes, regulations, and Medicaid policies across all 50 US states and the District of Columbia.

## Methods

This study followed EQUATOR Network guidance for transparent reporting. Medicaid enrollees as of August 2025 were included. Policies were coded using a structured codebook (eMethods in Supplement 1) on a 5-point scale—ranging from most to least supportive—across 3 domains (1) tele-buprenorphine for new patients, (2) tele-buprenorphine for established patients, and (3) availability of enhanced Medicaid reimbursement for tele-buprenorphine (eg, collaborative care for behavioral health integration, bundled office-based OUD care management, targeted case management, or opioid health home payments). We used the Center for Connected Health Policy database as the primary source for state policies^5^ and reviewed each state’s Medicaid policy manual to identify reimbursement provisions. Discrepancies were resolved during weekly meetings and through final review by the lead author (J.S.), and ratings were iteratively refined through team consensus, expert review (J.R., D.A.), and supplemental searches of publicly available statutes, regulations, and agency guidance. When policies conflicted, the more restrictive interpretation was applied. This analysis of publicly available policies did not involve human subjects or identifiable private information and was conducted as part of a study approved by the RAND institutional review board.

## Results

**Figure 1.**
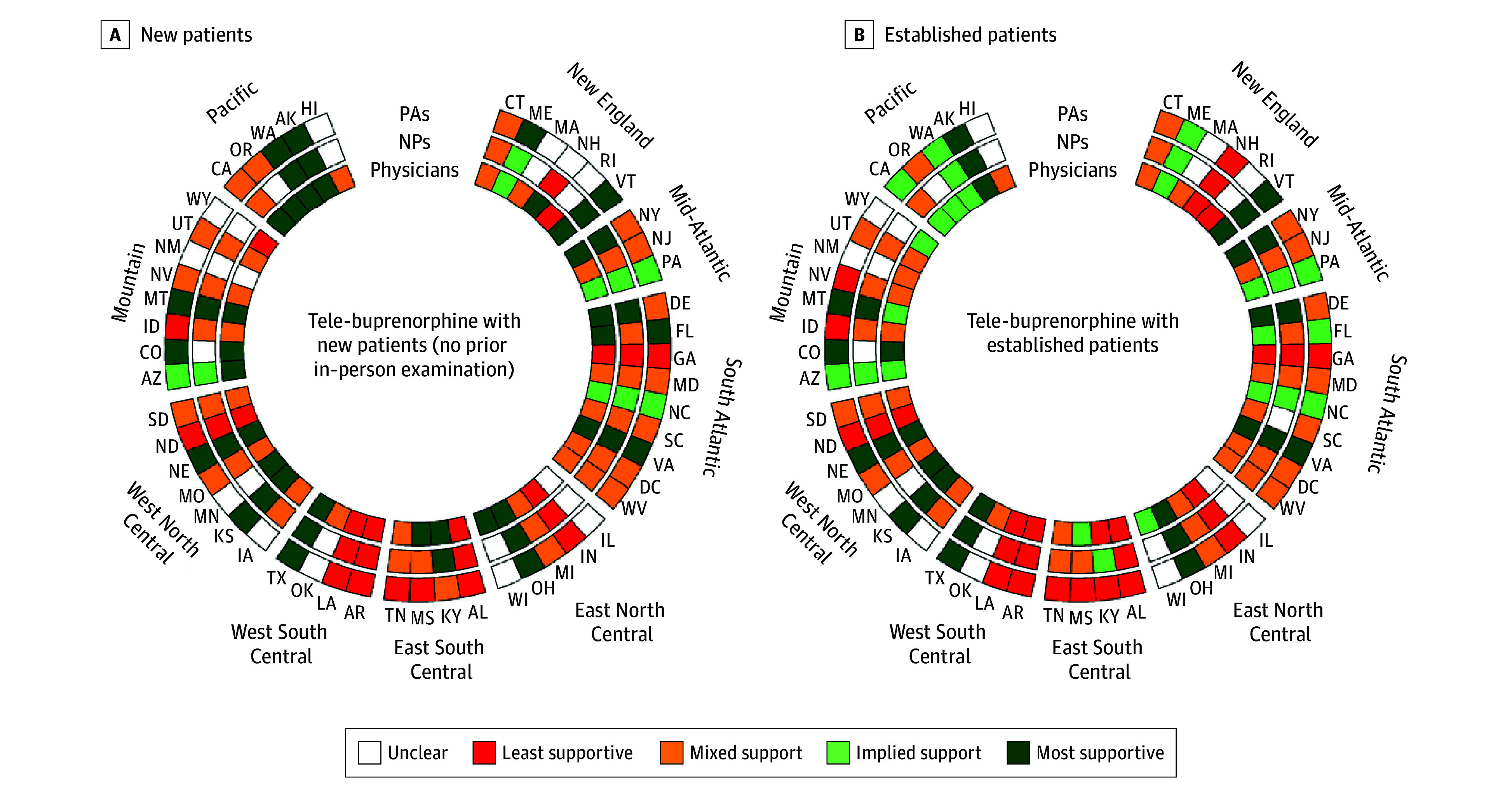
Radial Diagrams of State-Level Policy Support for Fully Virtual Tele-Buprenorphine With New and Established Patients With Medicaid Insurance, by Clinican Type (as of August 2025) Based on the analysis of publicly available legal and regulatory guidance on online prescribing and scope-of-practice laws. For ratings, most supportive connotes explicit support for tele-buprenorphine (with no in-person supervision or collaboration requirements for NPs or PAs), implied support indicates that tele-buprenorphine was neither disallowed nor explicitly supported by policy provisions, mixed support means that tele-buprenorphine was permitted if burdensome requirements are met, least supportive indicates an explicit restriction or prohibition of tele-buprenorphine (or in-person supervision or collaboration requirements for NPs or PAs), and unclear was used sparingly in cases in which there were no relevant state policies. NP indicates nurse practitioner; PA, physician assistant.

State policy support for fully virtual tele-buprenorphine care varied widely across clinician types (Figure 1). Six states prohibited all 3 clinician types from delivering tele-buprenorphine to new and established patients (Alabama, Arkansas, Georgia, Indiana, Louisiana, and North Dakota). Supportive policies were primarily concentrated in western states (eg, Alaska, Montana); restrictive policies were clustered in southern states (eg, Arkansas, Louisiana). Ten states had more supportive policies for tele-buprenorphine with new patients than for established patients, whereas only a few had the opposite pattern. Restrictions reflected a complex patchwork of policies and guidance (eTable in Supplement 1).

Policies for NPs and PAs were generally more restrictive or unclear than those for physicians (eg, Arizona, Mississippi, New Hampshire, Wisconsin, and Oregon). Multiple states mandated in-person supervision or in-state collaborating physicians (eg, Idaho, Nevada, Tennessee).

**Figure 2.**
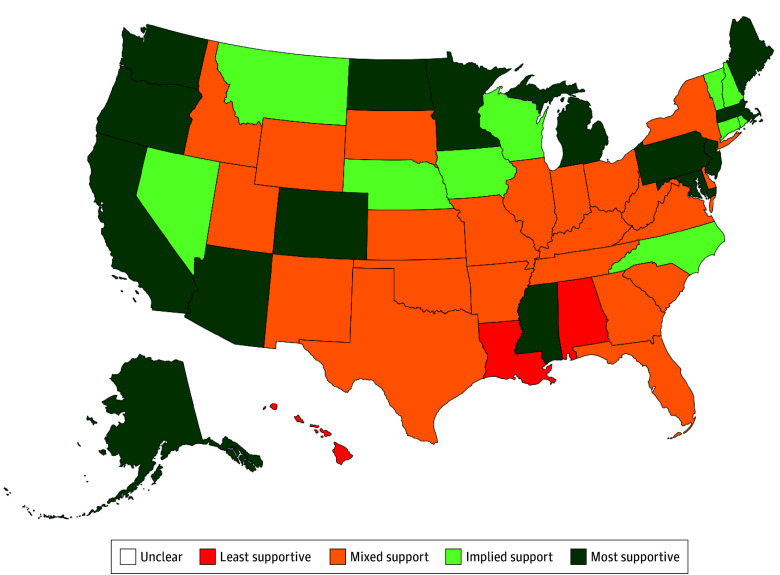
Map of State-Level Policy Support for Enhanced Medicaid Reimbursement for Fully Virtual Tele-Buprenorphine Prescribing to Patients With Medicaid Insurance (as of August 2025) Based on the analysis of publicly available Medicaid policies. For ratings, most supportive connotes an explicit allowance for enhanced Medicaid reimbursement (eg, codes for collaborative care, bundled office-based opioid use disorder care management, targeted case management, or opioid health home payments), implied support indicates an explicit allowance for some limited enhanced reimbursement (limited either in amount or by operational requirements), mixed support indicates that no enhanced reimbursement was available for tele-buprenorphine care but basic reimbursement was available, and least supportive indicates no Medicaid reimbursement for tele-buprenorphine.

Only 15 states’ policy environments supported enhanced Medicaid reimbursement for tele-buprenorphine, such as through targeted case management or collaborative care (Figure 2).

## Discussion

The proposed special registration pathway could maintain expanded OUD care access. However, the promise of federal reforms to expand access may go unrealized for clinicians facing state policy barriers, particularly NPs and PAs, who have become the dominant buprenorphine prescribers.^6^ Medicaid enrollees comprise 40% of adults with OUD, and enhanced reimbursement facilitates comprehensive OUD care delivery (eg, care navigation and after-hours support) that would otherwise involve uncompensated staff and clinician time.^7^ Study limitations include the interpretative nature of coding, the rapid evolution of telehealth policies, and the complexity of policies governing licensing, scope of practice, and reimbursement restrictions, which may not have been fully captured.

Some state policy restrictions may reflect intentional policy choices, whereas others may stem from laws predating the federal rule changes that may unintentionally restrict access. Reevaluating these policies in light of changing federal standards could help balance the need to mitigate diversion risk and ensure quality and safety with the benefits of expanding access to lifesaving OUD care.

## Notes

Supplement 1. eMethods. Rater Codebook eTable. Summary of State Policies Relevant to Fully Virtual Tele-Buprenorphine Care for Medicaid Enrollees, as of August 2025

Supplement 2. Data Sharing Statement
