# Supplementary material for: State Tele-Buprenorphine Prescribing Policies by Medical Professional Type
Source: JAMA Health Forum. 2026 Apr 24;7(4):e260420. doi: 10.1001/jamahealthforum.2026.0420 (PMC13109793; doi:10.1001/jamahealthforum.2026.0420)
Supplement: Supplement 1. — eMethods. Rater Codebook eTable. Summary of State Policies Relevant to Fully Virtual Tele-Buprenorphine Care for Medicaid Enrollees, as of August 2025 [file jamahealthforum-e260420-s001.pdf]

Supplemental Online Content

Sousa JL, Landis RK, Senator B. Variation in state tele-buprenorphine policies by medical professional type. *JAMA Health Forum*. 2026;7(4):e260420. doi:10.1001/jamahealthforum.2026.0420

eMethods. Rater Codebook
eTable. Summary of State Policies Relevant to Fully Virtual Tele-Buprenorphine Care for Medicaid Enrollees, as of August 2025

This supplemental material has been provided by the authors to give readers additional information about their work.

© 2026 Sousa JL, et al. *JAMA Health Forum*.

eMethods. Rater Codebook

Research Questions

1. To what extent does the state policy environment support, restrict, or prohibit/prevent an provider prescribing of buprenorphine for OUD care via telemedicine to ***new*** patients without an in-person exam or visit? [RQ1]
2. To what extent does the state policy environment support, restrict, or prohibit/prevent providers prescribing of buprenorphine to ***established*** patients[1] for ***ongoing/established*** OUD care via telemedicine in a fully virtual care model? [RQ2]?
3. To what extent does the state ***Medicaid*** agency support, restrict, or deny reimbursement to providers for BUP prescribing ***to new and established*** patients via telemedicine in a fully virtual care model? [RQ3]

Response options:

Most supportive	Explicit support
Implied support	Neither disallowed/prohibited nor explicitly supported
Mixed support	Permitted if burdensome requirements are met
Least supportive	Explicit restriction or prohibition
Unclear (use this sparingly)	No relevant policies

[1] Established patients are defined as those with whom the provider has an established patient-provider relationship.

© 2026 Sousa JL, et al. *JAMA Health Forum*.

Codebooks

Table 1. Codebook for Ratings on State Policies on Virtual Prescribing of Buprenorphine for OUD Care by MDs

#	Rating	Definition
RQ1-MDs	Most supportive	All 4 conditions must be met:<br>• Explicit support for **<u>at least one</u>** of the following:<br>o Establishing a patient-provider or patient-physician relationship via telemedicine **OR**<br>o Initial or any prescribing of controlled substances or BUP by physicians or providers via telemedicine **AND**<br>• No explicit prohibition (or no relevant policies) on **<u>ANY</u>** of the following:<br>o Establishing the patient-provider or patient-physician relationship via telemedicine without an in-person exam **OR**<br>o Initial or any physician or provider prescribing of controlled substances or BUP **AND**<br>• No requirement of **<u>ANY</u>** of any prohibitive (i.e., in-person) activities prior to the use of telemedicine to establish the patient-provider or patient-physician relationship or for physician or provider prescribing of controlled substances (e.g., in-person exam) **AND**<br>• No burdensome requirements (see 'Mixed' for examples).
	Implied support	All 5 conditions must be met:<br>• No explicit support for **<u>either</u>** of the following:<br>o Establishing a patient-provider relationship or patient-physician via telemedicine **OR**<br>o Initial or any prescribing of controlled substances or BUP via telemedicine **AND**<br>• No explicit prohibition (or no relevant policies) on **<u>ANY</u>** of the following:<br>o Establishing the patient-provider or patient-physician relationship via telemedicine without an in-person exam **OR**<br>o Initial or any physician or provider prescribing of controlled substances or BUP **AND**<br>• No requirement of **<u>ANY</u>** of any prohibitive (i.e., in-person) activities prior to the use of telemedicine to establish the patient-provider or patient-physician relationship or for physician or provider prescribing of controlled substances (e.g., in-person exam) **AND**<br>• No burdensome requirements (see 'Mixed' for examples), **AND**<br>• Something that makes the rater feel that MD or provider initial prescribing of controlled substances/BUP with new patients is permitted.

© 2026 Sousa JL, et al. *JAMA Health Forum*.

#	Rating	Definition
RQ1-MDs (contd)	Mixed support	All 3 conditions must be met:<br>• No explicit prohibition (or no relevant policies) on **ANY** of the following:<br>○ Establishing the patient-provider relationship or patient-physician via telemedicine without an in-person exam OR<br>○ Initial or any physician or provider prescribing of controlled substances or BUP **AND**<br>• No requirement of **ANY** of any prohibitive (i.e., in-person) activities prior to the use of telemedicine to establish the patient-provider or patient-physician relationship or for physician or provider prescribing of controlled substances (e.g., in-person exam) **AND**<br>• Any particularly burdensome (i.e., difficult or time-consuming) requirements as preconditions for physician or provider initial or any virtual BUP prescribing (or BUP prescribing in general) OR for establishing a patient-provider or patient-physician relationship via telemedicine.
	Least supportive	1 or more of the following conditions must be met:<br>• Explicit prohibition of **ANY** of the following:<br>○ Establishing the patient-provider or patient-physician relationship via telemedicine without an in-person exam **OR**<br>○ Initial or any prescribing of controlled substances or BUP **OR**<br>• Requirement of **ANY** of any prohibitive (i.e., in-person) activities prior to the use of telemedicine to establish the patient-provider or patient-physician relationship or for physician or provider prescribing of controlled substances:<br>○ An in-person physical exam or in-person verification of the patient's identity **OR**<br>○ Originating site restrictions (e.g., patient must go to a clinic or hospital for the first telehealth visit), **OR**<br>○ Distant site restrictions (e.g., provider must be in-person at a clinic or hospital during telehealth visit, or the provider must be located in the same state as the patient).
	Unclear	No relevant legal/regulatory support, restriction(s), or requirements(s) applicable to physicians or providers

© 2026 Sousa JL, et al. *JAMA Health Forum*.

#	Rating	Definition
RQ2-MDs	Most supportive	All 4 conditions must be met:<br>• Explicit support for any MD or provider prescribing of controlled substances or BUP via telemedicine **AND**<br>• No explicit prohibition (or no relevant policies) on **<u>ANY</u>** of the following:<br>○ Physician or provider prescribing of controlled substances or BUP via telemedicine **OR**<br>○ Physician or provider prescribing medication (in general) via telemedicine **AND**<br>• No requirement of **<u>ANY</u>** of any prohibitive (i.e., in-person) activities prior to the use of telemedicine to establish the patient-provider or patient-physician relationship or for physician or provider prescribing of controlled substances (e.g., in-person exam) **AND**<br>• No burdensome requirements (see 'Mixed support' for examples).
	Implied support	All 5 conditions must be met:<br>• No explicit support for MD or provider prescribing of controlled substances or BUP via telemedicine **AND**<br>• No explicit prohibition (or no relevant policies) on **<u>ANY</u>** of the following:<br>○ Physician or provider prescribing of controlled substances or BUP via telemedicine **OR**<br>○ Physician or provider prescribing medication (in general) via telemedicine **AND**<br>• No requirement of **<u>ANY</u>** of any prohibitive (i.e., in-person) activities prior to the use of telemedicine to establish the patient-provider or patient-physician relationship or for physician or provider prescribing of controlled substances (e.g., in-person exam) **AND**<br>• No burdensome "hoops" or requirements (see 'Mixed support' for examples), **AND**<br>• Something that makes the rater feel that MD or provider prescribing of controlled substances/BUP with established patients via telemedicine is permitted.
RQ2-MDs (contd)	Mixed support	All 3 conditions must be met:<br>• No explicit prohibition (or no relevant policies) on **<u>ANY</u>** of the following:<br>○ Physician or provider prescribing of controlled substances or BUP via telemedicine **OR**<br>○ Physician or provider prescribing medication (in general) via telemedicine **AND**<br>• No requirement of **<u>ANY</u>** of the following for MD or provider telemedicine prescribing of controlled substances/BUP with established patients (e.g., in-person exam) **AND**<br>• Any particularly burdensome (i.e., difficult or time-consuming) requirements as preconditions for virtual BUP prescribing (or BUP prescribing in general) with established patients (e.g., annually or within 6 months of initiating teleBUP).

© 2026 Sousa JL, et al. *JAMA Health Forum*.

#	Rating	Definition
	Least supportive	1 or more of the following conditions must be met:<br>• Explicit prohibition of **<u>ANY</u>** of the following:<br>○ Physician or provider prescribing of controlled substances or BUP via telemedicine **OR**<br>○ Physician or provider prescribing medication (in general) via telemedicine **AND**<br>• Requirement of **<u>ANY</u>** of any prohibitive (i.e., in-person) requirements as a precondition for physician or provider use of telemedicine to prescribe controlled substances or BUP (or any medication) to established patients, such as:<br>○ In-person visits (e.g., every few months or every year), **OR**<br>○ Originating site restrictions (e.g., patient must go to a clinic or hospital for telehealth visit), **OR**<br>○ Distant site restrictions (e.g., provider must be in-person at a clinic or hospital during telehealth visit, or the provider must be located in the same state as the patient).
	Unclear	No relevant legal/regulatory support, restriction(s), or requirements(s) applicable to MDs or providers

© 2026 Sousa JL, et al. *JAMA Health Forum*.

<table>
<tr><th>#</th><th>Rating</th><th>Definition</th></tr>
<tr><td rowspan="2">RQ3-MDs</td><td>Most supportive</td><td>All 3 conditions must be met:
Explicit allowance for <u>enhanced</u> Medicaid reimbursement for physician or provider telemedicine encounters that include prescribing of controlled substances or BUP (either in general or by telehealth/telemedicine in particular) AND
No explicit prohibition against (or no relevant policies on) <u>enhanced</u> Medicaid reimbursement for <u>ANY</u> of the following:
Establishing the patient-provider relationship via telemedicine without an in-person exam OR
Initial or any prescribing of controlled substances or BUP OR
Prescribing medication (in general) via telemedicine AND
No requirement of <u>ANY</u> of the following ‘deal-breakers’ as a condition for <u>enhanced</u> Medicaid reimbursement:
In-person exam or visits (e.g., before prescribing or every few months or every year), OR
Originating site restrictions (e.g., patient must go to a clinic or hospital for telehealth visit), OR
Distant site restrictions (e.g., provider must be in-person at a clinic or hospital during telehealth visit)</td></tr>
<tr><td>Implied support</td><td>All 3 conditions must be met:
Explicit allowance for some <u>limited enhanced</u> reimbursement (i.e., limited either in amount or by operational requirements) for physician or provider telemedicine encounters that include prescribing of controlled substances or BUP AND
No explicit prohibition against (or no relevant policies on) <u>limited enhanced</u> Medicaid reimbursement for <u>ANY</u> of the following:
Establishing the patient-provider relationship via telemedicine without an in-person exam OR
Initial or any prescribing of controlled substances or BUP OR
Prescribing medication (in general) via telemedicine AND
No requirement of <u>ANY</u> of the following for <u>limited enhanced</u> Medicaid reimbursement:
In-person exam or visits (e.g., before prescribing or every few months or every year), OR
Originating site restrictions (e.g., patient must go to a clinic or hospital for telehealth visit), OR
Distant site restrictions (e.g., provider must be in-person at a clinic or hospital during telehealth visit)</td></tr>
</table>

© 2026 Sousa JL, et al. *JAMA Health Forum*.

#	Rating	Definition
RQ3-MDs (contd)	Mixed support	All 3 conditions must be met:<br>• No enhanced reimbursement for virtual MOUD prescribing, but explicit allowance for **basic** FFS Medicaid reimbursement for physician or provider telemedicine encounters that include prescribing of controlled substances or BUP **AND**<br>• No explicit prohibition against providing **basic** FFS Medicaid reimbursement for **ANY** of the following:<br>○ Establishing the patient-provider relationship via telemedicine without an in-person exam **OR**<br>○ Initial or any prescribing of controlled substances or BUP **OR**<br>○ Prescribing medication (in general) via telemedicine **AND**<br>• No requirement of **ANY** of the following for **basic** FFS Medicaid reimbursement:<br>○ In-person exam or visits (e.g., before prescribing or every few months or every year), **OR**<br>○ Originating site restrictions (e.g., patient must go to a clinic or hospital for telehealth visit), **OR**<br>○ Distant site restrictions (e.g., provider must be in-person at a clinic or hospital during telehealth visit)
	Least supportive	1 or more of the following conditions must be met:<br>• No Medicaid reimbursement for MD or provider prescribing of controlled substances or BUP via telemedicine encounter<br>• Explicit prohibition against providing Medicaid reimbursement for **ANY** of the following:<br>○ Establishing the patient-provider relationship via telemedicine without an in-person exam **OR**<br>○ Prescribing of controlled substances or BUP via telemedicine **OR**<br>○ Prescribing medication (in general) via telemedicine **AND**<br>• Explicit requirement of **ANY** of the following for Medicaid reimbursement:<br>○ In-person exam or visits (e.g., before prescribing or every few months or every year), **OR**<br>○ Originating site restrictions (e.g., patient must go to a clinic or hospital for telehealth visit), **OR**<br>○ Distant site restrictions (e.g., provider must be in-person at a clinic or hospital during telehealth visit, or the provider must be located in the same state as the patient).
	Unclear	No relevant legal/regulatory support, restriction(s), or requirements(s) applicable to physicians or providers

© 2026 Sousa JL, et al. *JAMA Health Forum*.

Table 2. Codebook for Ratings on State Policies on Virtual Prescribing of Buprenorphine for OUD Care by NPs

#	Rating	Definition
RQ1 – NPs	Most supportive	All 4 conditions must be met:<br>• Explicit support for **at least one** of the following:<br>○ Establishing a patient-provider or patient-NP relationship via telemedicine **OR**<br>○ Initial or any prescribing of controlled substances or BUP by NPs or providers via telemedicine **AND**<br>• No explicit prohibition (or no relevant policies) on **ANY** of the following:<br>○ Establishing the patient-provider or patient-NP relationship via telemedicine without an in-person exam **OR**<br>○ Initial or any NP or provider prescribing of controlled substances or BUP **AND**<br>• No requirement of **ANY** of any prohibitive (e.g., in-person) activities prior to the use of telemedicine to establish the patient-provider or patient-NP relationship or for NP or provider prescribing of controlled substances (e.g., in-person exam; in-person supervision) **AND**<br>• No burdensome requirements (see 'Mixed support' for examples).
	Implied support	All 5 conditions must be met:<br>• No explicit support for **either** of the following:<br>○ Establishing a patient-provider or patient-NP relationship via telemedicine **OR**<br>○ Initial or any prescribing of controlled substances or BUP by NPs or providers via telemedicine **AND**<br>• No explicit prohibition (or no relevant policies) on **ANY** of the following:<br>○ Establishing the patient-provider or patient-NP relationship via telemedicine without an in-person exam **OR**<br>○ Initial or any NP or provider prescribing of controlled substances or BUP **AND**<br>• No requirement of **ANY** of any prohibitive (e.g., in-person) activities prior to the use of telemedicine to establish the patient-provider or patient-NP relationship or for NP or provider prescribing of controlled substances (e.g., in-person exam; in-person supervision) **AND**<br>• No burdensome requirements (see 'Mixed support' for examples), **AND**<br>• Something that makes the rater feel that NP or provider initial prescribing of controlled substances/BUP with new patients is permitted.

© 2026 Sousa JL, et al. *JAMA Health Forum*.

<table>
<tr><th>#</th><th>Rating</th><th>Definition</th></tr>
<tr><td rowspan="3">RQ1 – NPs (cont.)</td><td>Mixed support</td><td>All 3 conditions must be met:
• No explicit prohibition (or no relevant policies) on <u>ANY</u> of the following:
  ○ Establishing the patient-provider or patient-NP relationship via telemedicine without an in-person exam OR
  ○ Initial or any NP or provider prescribing of controlled substances or BUP AND
• No requirement of <u>ANY</u> of any prohibitive (e.g., in-person) activities prior to the use of telemedicine to establish the patient-provider or patient-NP relationship or for NP or provider prescribing of controlled substances (e.g., in-person exam; in-person supervision) AND
• Any particularly burdensome (i.e., difficult or time-consuming) requirements for NPs as preconditions for NP or provider initial or any virtual BUP prescribing (or BUP prescribing in general) or for establishing a patient-provider or patient-NP relationship via telemedicine, such as:
  ○ Supervision requirements – burdensome supervision requirements, such an imposed maximum of 5 supervisees or fewer per supervising physician, or requiring frequent remote site visits or chart review for virtual buprenorphine prescribing by NPs. Note that supervision requirements should not count as a “hoop” unless they are particularly difficult or time-consuming, particularly relative to in-person supervision requirements.</td></tr>
<tr><td>Least supportive</td><td>1 or more of the following conditions must be met:
• Geographic issue that requires a physical presence (either between NP and MD or within the state)
• Explicit prohibition of <u>ANY</u> of the following:
  ○ Establishing the patient-provider or patient-NP relationship via telemedicine without an in-person exam OR
  ○ Initial or any NP or provider prescribing of controlled substances or BUP
• Requirement of <u>ANY</u> of any prohibitive (i.e., in-person) activities prior to the use of telemedicine to establish the patient-provider or patient-NP relationship or for NP or provider prescribing of controlled substances:
  ○ In-person supervision or collaboration requirements[2] OR
  ○ An in-person physical exam or in-person verification of the patient’s identity OR
  ○ Originating site restrictions (e.g., patient must go to a clinic or hospital for the first telehealth visit), OR
  ○ Distant site restrictions (e.g., provider must be in-person at a clinic or hospital during telehealth visit, or the provider must be located in the same state as the patient).</td></tr>
<tr><td>Unclear</td><td>No relevant legal/regulatory support, restriction(s), or requirements(s) about APRNs, NPs, advanced practice providers, or health care practitioners/providers generally</td></tr>
</table>

[2] Disregard any in-person supervision or collaboration requirements that apply only to new NPs (e.g., new graduates or first-time licensees).

© 2026 Sousa JL, et al. *JAMA Health Forum*.

#	Rating	Definition
RQ2 – NPs	Most supportive	All 4 conditions must be met:<br>• Explicit support for any NP or provider prescribing of controlled substances or BUP via telemedicine **AND**<br>• No explicit prohibition (or no relevant policies) on **<u>ANY</u>** of the following:<br>○ NP or provider prescribing of controlled substances or BUP via telemedicine **OR**<br>○ NP or provider prescribing medication (in general) via telemedicine **AND**<br>• No requirement of **<u>ANY</u>** of any prohibitive (i.e., in-person) activities prior to the use of telemedicine to establish the patient-provider or patient-NP relationship or for NP or provider prescribing of controlled substances (e.g., in-person exam; in-person supervision; see 'Least Supportive' for examples) **AND**<br>• No burdensome requirements ('Mixed' for examples).
	Implied support	All 5 conditions must be met:<br>• No explicit support for prescribing of controlled substances or BUP via telemedicine **AND**<br>• No explicit prohibition (or no relevant policies) on **<u>ANY</u>** of the following:<br>○ NP or provider prescribing of controlled substances or BUP via telemedicine **OR**<br>○ NP or provider prescribing medication (in general) via telemedicine **AND**<br>• No requirement of **<u>ANY</u>** of any prohibitive (i.e., in-person) activities prior to the use of telemedicine to establish the patient-provider or patient-NP relationship or for NP or provider prescribing of controlled substances (e.g., in-person exam; in-person supervision; see 'Least Supportive' for examples) **AND**<br>• No burdensome requirements (see 'Mixed' for examples), **AND**<br>• Something that makes the rater feel that PA or provider prescribing of controlled substances/BUP with established patients via telemedicine is permitted.

© 2026 Sousa JL, et al. *JAMA Health Forum*.

#	Rating	Definition
RQ2 – NPs (contd)	Mixed support	All 3 conditions must be met:<br>• No explicit prohibition (or no relevant policies) on **ANY** of the following:<br>o NP or provider prescribing of controlled substances or BUP via telemedicine **OR**<br>o NP or provider prescribing medication (in general) via telemedicine **AND**<br>• No requirement of **ANY** of any prohibitive (i.e., in-person) activities prior to the use of telemedicine to establish the patient-provider or patient-NP relationship or for NP or provider prescribing of controlled substances (e.g., in-person exam; in-person supervision; see ‘Least Supportive’ for examples) **AND**<br>• Any particularly burdensome (i.e., difficult or time-consuming) “hoops” or requirements for NPs as preconditions for virtual BUP prescribing (or BUP prescribing in general with established patients (e.g., annually or within 6 months of establishing a patient-provider relationship via telemedicine), such as:<br>o **Supervision requirements** – burdensome supervision requirements, such an imposed maximum of 5 supervisees or fewer per supervising physician, or requiring frequent remote site visits or chart review for virtual buprenorphine prescribing by NPs. Note that supervision requirements should not count as a “hoop” unless they are particularly difficult or time-consuming, particularly relative to in-person supervision requirements.
	Least supportive	1 or more of the following conditions must be met:<br>• Explicit prohibition of **ANY** of the following:<br>o NP or provider prescribing of controlled substances or BUP via telemedicine **OR**<br>o NP or provider prescribing medication (in general) via telemedicine **AND**<br>• Requirement of **ANY** of any prohibitive (i.e., in-person) activities prior to the use of telemedicine to establish the patient-provider or patient-NP relationship or for NP or provider prescribing of controlled substances, such as:<br>o In-person supervision or collaboration requirements[3] **OR**<br>o In-person visits (e.g., every few months or every year), **OR**<br>o Originating site restrictions (e.g., patient must go to a clinic or hospital for telehealth visit), **OR**<br>o Distant site restrictions (e.g., provider must be in-person at a clinic or hospital during telehealth visit, or the provider must be located in the same state as the patient).
	Unclear	No relevant legal/regulatory support, restriction(s), or requirements(s) about APRNs, NPs, advanced practice providers, or health care practitioners/providers generally

[3] Disregard any in-person supervision or collaboration requirements that apply only to new NPs (e.g., new graduates or first-time licensees).

© 2026 Sousa JL, et al. *JAMA Health Forum*.

<table>
<tr><th>#</th><th>Rating</th><th>Definition</th></tr>
<tr><td rowspan="2">RQ3 – NPs</td><td>Most supportive</td><td>All 3 conditions must be met:
Explicit allowance for <u>enhanced</u> Medicaid reimbursement for telemedicine encounters that include NP or provider prescribing of controlled substances or BUP (either in general or by telehealth/telemedicine in particular) AND
No explicit prohibition against (or no relevant policies on) <u>enhanced</u> Medicaid reimbursement for <u>ANY</u> of the following:
Establishing the patient-provider relationship via telemedicine without an in-person exam OR
Initial or any prescribing of controlled substances or BUP OR
Prescribing medication (in general) via telemedicine AND
No requirement of <u>ANY</u> of the following for <u>enhanced</u> Medicaid reimbursement:
In-person exam or visits (e.g., before prescribing or every few months or every year), OR
Originating site restrictions (e.g., patient must go to a clinic or hospital for telehealth visit), OR
Distant site restrictions (e.g., provider must be in-person at a clinic or hospital during telehealth visit)</td></tr>
<tr><td>Implied support</td><td>All 3 conditions must be met:
Explicit allowance for some <u>limited enhanced</u> reimbursement (i.e., limited either in amount or by operational requirements) for NP or provider telemedicine encounters that include prescribing of controlled substances or BUP AND
No explicit prohibition against (or no relevant policies on) <u>limited enhanced</u> Medicaid reimbursement for <u>ANY</u> of the following:
Establishing the patient-provider relationship via telemedicine without an in-person exam OR
Initial or any prescribing of controlled substances or BUP OR
Prescribing medication (in general) via telemedicine AND
No requirement of <u>ANY</u> of the following for <u>limited enhanced</u> Medicaid reimbursement:
In-person exam or visits (e.g., before prescribing or every few months or every year), OR
Originating site restrictions (e.g., patient must go to a clinic or hospital for telehealth visit), OR
Distant site restrictions (e.g., provider must be in-person at a clinic or hospital during telehealth visit)</td></tr>
</table>

© 2026 Sousa JL, et al. *JAMA Health Forum*.

#	Rating	Definition
RQ3 – NPs (contd)	Mixed support	All 3 conditions must be met:<br>• No enhanced reimbursement for NP or provider virtual MOUD prescribing, but explicit allowance for **basic** FFS Medicaid reimbursement telemedicine encounters that include prescribing of controlled substances or BUP **AND**<br>• No explicit prohibition against providing **basic** FFS Medicaid reimbursement for **ANY** of the following:<br>o Establishing the patient-provider relationship via telemedicine without an in-person exam **OR**<br>o Initial or any prescribing of controlled substances or BUP **OR**<br>o Prescribing medication (in general) via telemedicine **AND**<br>• No requirement of **ANY** of the following for **basic** FFS Medicaid reimbursement:<br>o In-person exam or visits (e.g., before prescribing or every few months or every year), **OR**<br>o Originating site restrictions (e.g., patient must go to a clinic or hospital for telehealth visit), **OR**<br>o Distant site restrictions (e.g., provider must be in-person at a clinic or hospital during telehealth visit)
	Least supportive	1 or more of the following conditions must be met:<br>• No Medicaid reimbursement for NP or provider prescribing of controlled substances or BUP via telemedicine encounter<br>• Explicit prohibition against providing Medicaid reimbursement for **ANY** of the following:<br>o Establishing the patient-provider relationship via telemedicine without an in-person exam **OR**<br>o Prescribing of controlled substances or BUP via telemedicine **OR**<br>o Prescribing medication (in general) via telemedicine **AND**<br>• Explicit requirement of **ANY** of the following for Medicaid reimbursement:<br>o In-person exam or visits (e.g., before prescribing or every few months or every year), **OR**<br>o Originating site restrictions (e.g., patient must go to a clinic or hospital for telehealth visit), **OR**<br>o Distant site restrictions (e.g., provider must be in-person at a clinic or hospital during telehealth visit, or the provider must be located in the same state as the patient).
	Unclear	No relevant legal/regulatory support, restriction(s), or requirements(s) about APRNs, NPs, advanced practice providers, or health care practitioners/providers generally

© 2026 Sousa JL, et al. *JAMA Health Forum*.

Table 3. Codebook for Ratings on State Policies on Virtual Prescribing of Buprenorphine for OUD Care by PAs

#	Rating	Definition
RQ1 – PAs	Most supportive	All 4 conditions must be met:<br>• Explicit support for **at least one** of the following:<br>○ Establishing a patient-provider or patient-PA relationship via telemedicine **OR**<br>○ Initial or any PA or provider prescribing of controlled substances or BUP via telemedicine **AND**<br>• No explicit prohibition (or no relevant policies) on **ANY** of the following:<br>○ Establishing the patient-provider or patient-PA relationship via telemedicine without an in-person exam **OR**<br>○ Initial or any PA or provider prescribing of controlled substances or BUP **AND**<br>• No requirement of **ANY** of any prohibitive (e.g., in-person) activities prior to the use of telemedicine to establish the patient-provider or patient-PA relationship or for PA or provider prescribing of controlled substances (e.g., in-person exam; in-person supervision; see 'Least Supportive' for examples) **AND**<br>• No burdensome requirements (see 'Mixed' for examples).
	Implied support	All 5 conditions must be met:<br>• No explicit support for **either** of the following:<br>○ Establishing a patient-provider or patient-PA relationship via telemedicine **OR**<br>○ Initial or any PA or provider prescribing of controlled substances or BUP via telemedicine **AND**<br>• No explicit prohibition (or no relevant policies) on **ANY** of the following:<br>○ Establishing the patient-provider or patient-PA relationship via telemedicine without an in-person exam **OR**<br>○ Initial or any PA or provider prescribing of controlled substances or BUP **AND**<br>• No requirement of **ANY** of any prohibitive (e.g., in-person) activities prior to the use of telemedicine to establish the patient-provider or patient-PA relationship or for PA or provider prescribing of controlled substances (e.g., in-person exam; in-person supervision; see 'Least Supportive' for examples) **AND**<br>• No burdensome requirements (see 'Mixed' for examples), **AND**<br>• Something that makes the rater feel that PA or provider initial prescribing of controlled substances/BUP with new patients is permitted.

© 2026 Sousa JL, et al. *JAMA Health Forum*.

#	Rating	Definition
RQ1 – PAs (cont.)	Mixed support	All 3 conditions must be met:<br>• No explicit prohibition (or no relevant policies) on **ANY** of the following:<br>○ Establishing the patient-provider or patient-PA relationship via telemedicine without an in-person exam OR<br>○ Initial or any PA or provider prescribing of controlled substances or BUP via telemedicine **AND**<br>• No requirement of **ANY** of any prohibitive (e.g., in-person) activities prior to the use of telemedicine to establish the patient-provider or patient-PA relationship or for PA or provider prescribing of controlled substances (e.g., in-person exam; in-person supervision; see 'Least Supportive' for examples) **AND**<br>• Any particularly burdensome (i.e., difficult or time-consuming) requirements for PA or provider initial or any virtual BUP prescribing (or BUP prescribing in general) or for establishing a patient-provider or patient-PA relationship via telemedicine, such as:<br>○ **Supervision requirements** – burdensome supervision requirements, such an imposed maximum of 5 supervisees or fewer per supervising physician, or requiring frequent remote site visits or chart review for virtual buprenorphine prescribing by PAs. Note that supervision requirements should not count as a "hoop" unless they are particularly difficult or time-consuming, particularly relative to in-person supervision requirements.
	Least supportive	1 or more of the following conditions must be met:<br>• Explicit prohibition of **ANY** of the following:<br>○ Establishing the patient-provider or patient-PA relationship via telemedicine without an in-person exam **OR**<br>○ Initial or any PA or provider prescribing of controlled substances or BUP via telemedicine **AND**<br>• No requirement of **ANY** of any prohibitive (e.g., in-person) activities for the use of telemedicine by PAs or providers to prescribe controlled substances or BUP (or any medication) or to establish the patient-provider or patient-NP relationship via telemedicine, such as<br>○ In-person supervision or collaboration requirements[4] **OR**<br>○ An in-person physical exam or in-person verification of the patient's identity **OR**<br>○ Originating site restrictions (e.g., patient must go to a clinic or hospital for the first telehealth visit), **OR**<br>○ Distant site restrictions (e.g., provider must be in-person at a clinic or hospital during telehealth visit, or the provider must be located in the same state as the patient).
	Unclear	No relevant legal/regulatory support, restriction(s), or requirements(s) about PAs, advanced practice providers, or health care practitioners/providers generally

[4] Disregard any in-person supervision or collaboration requirements that apply only to new PAs (e.g., new graduates or first-time licensees).

© 2026 Sousa JL, et al. *JAMA Health Forum*.

#	Rating	Definition
RQ2 – PAs	Most supportive	All 4 conditions must be met:<br>• Explicit support for any PA or provider prescribing of controlled substances or BUP via telemedicine **AND**<br>• No explicit prohibition (or no relevant policies) on **<u>ANY</u>** of the following:<br>o PA or provider prescribing of controlled substances or BUP via telemedicine **OR**<br>o PA or provider prescribing medication (in general) via telemedicine **AND**<br>• No requirement of **<u>ANY</u>** of any prohibitive (i.e., in-person) activities prior to the use of telemedicine to establish the patient-provider or patient-PA relationship or for PA or provider prescribing of controlled substances (e.g., in-person exam; in-person supervision; see ‘Least Supportive’ for examples) **AND**<br>• No burdensome requirements (see ‘Mixed’ for examples).
	Implied support	All 5 conditions must be met:<br>• No explicit support for PA or provider prescribing of controlled substances or BUP via telemedicine **AND**<br>• No explicit prohibition (or no relevant policies) on **<u>ANY</u>** of the following:<br>o PA or provider prescribing of controlled substances or BUP via telemedicine **OR**<br>o PA or provider prescribing medication (in general) via telemedicine **AND**<br>• No requirement of **<u>ANY</u>** of any prohibitive (i.e., in-person) activities prior to the use of telemedicine to establish the patient-provider or patient-PA relationship or for PA or provider prescribing of controlled substances (e.g., in-person exam; in-person supervision; see ‘Least Supportive’ for examples) **AND**<br>• No burdensome requirements (see ‘Mixed’ for examples), **AND**<br>• Something that makes the rater feel that PA or provider prescribing of controlled substances/BUP with established patients via telemedicine is permitted.

© 2026 Sousa JL, et al. *JAMA Health Forum*.

#	Rating	Definition
RQ2 – PAs (contd)	Mixed support	All 3 conditions must be met:<br>• No explicit prohibition (or no relevant policies) on **ANY** of the following:<br>○ PA or provider prescribing of controlled substances or BUP via telemedicine **OR**<br>○ PA or provider prescribing medication (in general) via telemedicine **AND**<br>• No requirement of **ANY** of the following for PA or provider telemedicine prescribing of controlled substances/BUP with established patients (e.g., in-person exam; in-person supervision; see 'Least Supportive' for examples) **AND**<br>• Any particularly burdensome (i.e., difficult or time-consuming) requirements for PAs as preconditions for virtual BUP prescribing (or BUP prescribing in general with established patients (e.g., annually or within 6 months of establishing a patient-provider relationship via telemedicine), such as:<br>○ **Supervision requirements** – burdensome supervision requirements, such an imposed maximum of 5 supervisees or fewer per supervising physician, or requiring frequent remote site visits or chart review for virtual buprenorphine prescribing by PAs. Note that supervision requirements should not count as a "hoop" unless they are particularly difficult or time-consuming, particularly relative to in-person supervision requirements.
	Least supportive	1 or more of the following conditions must be met:<br>• Explicit prohibition of **ANY** of the following:<br>○ PA or provider prescribing of controlled substances or BUP via telemedicine **OR**<br>○ PA or provider prescribing medication (in general) via telemedicine<br>• No requirement of **ANY** of any prohibitive (e.g., in-person) activities as a precondition for PA or provider use of telemedicine to prescribe controlled substances or BUP (or any medication) to established patients, such as:<br>○ In-person supervision or collaboration requirements[5] **OR**<br>○ In-person visits (e.g., every few months or every year), **OR**<br>○ Originating site restrictions (e.g., patient must go to a clinic or hospital for telehealth visit), **OR**<br>○ Distant site restrictions (e.g., provider must be in-person at a clinic or hospital during telehealth visit, or the provider must be located in the same state as the patient).
	Unclear	No relevant legal/regulatory support, restriction(s), or requirements(s) about PAs, advanced practice providers, or health care practitioners/providers generally

[5] Disregard any in-person supervision or collaboration requirements that apply only to new PAs (e.g., new graduates or first-time licensees).

© 2026 Sousa JL, et al. *JAMA Health Forum*.

<table>
<tr><th>#</th><th>Rating</th><th>Definition</th></tr>
<tr><td rowspan="2">RQ3 – PAs</td><td>Most supportive</td><td>All 3 conditions must be met:

Explicit allowance for <u>enhanced</u> Medicaid reimbursement for telemedicine encounters that include PA or provider prescribing of controlled substances or BUP (either in general or by telehealth/telemedicine in particular) AND
No explicit prohibition against (or no relevant policies on) <u>enhanced</u> Medicaid reimbursement for <u>ANY</u> of the following:

Establishing the patient-provider relationship via telemedicine without an in-person exam OR
Initial or any prescribing of controlled substances or BUP OR
Prescribing medication (in general) via telemedicine AND

No requirement of <u>ANY</u> of the following prohibitive requirements as a condition for <u>enhanced</u> Medicaid reimbursement:

In-person exam or visits (e.g., before prescribing or every few months or every year), OR
Originating site restrictions (e.g., patient must go to a clinic or hospital for telehealth visit), OR
Distant site restrictions (e.g., provider must be in-person at a clinic or hospital during telehealth visit)

</td></tr>
<tr><td>Implied support</td><td>All 3 conditions must be met:

Explicit allowance for some <u>limited enhanced</u> reimbursement (i.e., limited either in amount or by operational requirements) for telemedicine encounters that include prescribing of controlled substances or BUP AND
No explicit prohibition against (or no relevant policies on) <u>limited enhanced</u> Medicaid reimbursement for <u>ANY</u> of the following:

Establishing the patient-provider relationship via telemedicine without an in-person exam OR
Initial or any prescribing of controlled substances or BUP OR
Prescribing medication (in general) via telemedicine AND

No requirement of <u>ANY</u> of the following for <u>limited enhanced</u> Medicaid reimbursement:

In-person exam or visits (e.g., before prescribing or every few months or every year), OR
Originating site restrictions (e.g., patient must go to a clinic or hospital for telehealth visit), OR
Distant site restrictions (e.g., provider must be in-person at a clinic or hospital during telehealth visit), AND

</td></tr>
</table>

© 2026 Sousa JL, et al. *JAMA Health Forum*.

#	Rating	Definition
RQ3 – PAs (contd)	Mixed support	All 3 conditions must be met:<br>• No enhanced reimbursement for virtual MOUD prescribing, but explicit allowance for **basic** FFS Medicaid reimbursement telemedicine encounters that include prescribing of controlled substances or BUP **AND**<br>• No explicit prohibition against providing **basic** FFS Medicaid reimbursement for **ANY** of the following:<br>o Establishing the patient-provider relationship via telemedicine without an in-person exam **OR**<br>o Initial or any prescribing of controlled substances or BUP **OR**<br>o Prescribing medication (in general) via telemedicine **AND**<br>• No requirement of **ANY** of the following for **basic** FFS Medicaid reimbursement:<br>o In-person exam or visits (e.g., before prescribing or every few months or every year), **OR**<br>o Originating site restrictions (e.g., patient must go to a clinic or hospital for telehealth visit), **OR**<br>o Distant site restrictions (e.g., provider must be in-person at a clinic or hospital during telehealth visit)
	Least supportive	1 or more of the following conditions must be met:<br>• No Medicaid reimbursement for PA or provider prescribing of controlled substances or BUP via telemedicine encounter<br>• Explicit prohibition against providing Medicaid reimbursement for **ANY** of the following:<br>o Establishing the patient-provider relationship via telemedicine without an in-person exam **OR**<br>o Prescribing of controlled substances or BUP via telemedicine **OR**<br>o Prescribing medication (in general) via telemedicine **AND**<br>• Explicit requirement of **ANY** of the following for Medicaid reimbursement:<br>o In-person exam or visits (e.g., before prescribing or every few months or every year), **OR**<br>o Originating site restrictions (e.g., patient must go to a clinic or hospital for telehealth visit), **OR**<br>o Distant site restrictions (e.g., provider must be in-person at a clinic or hospital during telehealth visit, or the provider must be located in the same state as the patient).
	Unclear	No relevant legal/regulatory support, restriction(s), or requirements(s) about PAs, advanced practice providers, or health care practitioners/providers generally

© 2026 Sousa JL, et al. *JAMA Health Forum*.

Coding Guidance

Applicable to all ratings/all tables:

- Do not include in your analysis guidance or policies about the following:
  - Prescribing of controlled substances in other schedules (e.g., I, II, IV, V) and of buprenorphine for pain management
  - One-time prescriptions or temporary care (e.g., covering for another provider) and special clauses for medical emergencies
  - Osteopathic physician and pharmacist prescribers
  - Continuing education
  - In-state licensure
- Modality – assume the provider and patient are meeting for synchronous video telehealth visits, and disregard any policies about audio-only visits.
- Originating site – assume the patient is located in the home during the visit.
- Distant site – assume the provider is located in their home during the visit, and assume they are based in a different U.S. state than that of the patient.
- The following requirements should be noted in the 'Explanation' field if applicable, but should NOT be counted as "hoops" warranting a rating of 'Mixed Support' unless they are unusually difficult or time-consuming:
  - **Controlled substance registration** – requiring the provider to register with the state controlled substance authority
  - **Licensure** – requiring that the physician be licensed in the state where the patient is located (i.e., the originating site).
  - **MAT/MOUD program –** requiring a special MOUD license (e.g., which limits the number of patients the prescriber can treat or mandates use of a prescription drug monitoring program).
  - **Telehealth registration** – requiring telehealth registration as an alternative or in addition to in-state medical licensure.

Additional Guidance on Terminology

- Watch for references to buprenorphine and OUD care using the following terms:
  - "Buprenorphine," "buprenorphine/naloxone," or "suboxone,"
  - "Schedule III controlled substance," "controlled substance," or "controlled medication," and
  - "MOUD" (medications for opioid use disorder), "SUD" (substance use disorder), "MAT" (medication assisted treatment), "OBOT" (office-based opioid treatment), or "OBMAT" (office-based medication-assisted treatment).
- In analyzing policies on online prescribing or telehealth, we're including those applicable to synchronous (real-time) visits via video between a prescriber (MD, NP, or PA) and a patient. These might be described using the following terms:
  - "Online prescribing," "remote prescribing," or "virtual prescribing,"
  - "Telemedicine," "telehealth," or "virtual" care, and
  - Combinations of these terms, sometimes in conjunction with terms for BUP/MOUD– for example:
  - "Telebuprenorphine" or "tele-BUP,"
  - "Virtual MOUD," "teleMOUD" / "tele-MOUD," or "teleMAT", and
  - "Telebehavioral health," "teleBH," or "teleSUD."
- Also watch for references to the following terms, which may be used in lieu of other terminology:
  - "Induction" or "initiation" of buprenorphine
  - "Patient-provider relationship" or "patient-physician relationship" and
  - "Maintenance prescribing" or "continuation"/ "continuous" prescribing.

© 2026 Sousa JL, et al. *JAMA Health Forum*.
