# Supplementary material for: State Tele-Buprenorphine Prescribing Policies by Medical Professional Type
Source: JAMA Health Forum. 2026 Apr 24;7(4):e260420. doi: 10.1001/jamahealthforum.2026.0420 (PMC13109793; doi:10.1001/jamahealthforum.2026.0420)
Supplement: Supplement 2. — Data Sharing Statement [file jamahealthforum-e260420-s002.pdf]

Data Sharing Statement

Sousa. State Tele-Buprenorphine Prescribing Policies by Medical Professional Type. *JAMA Health Forum*. Published April 24, 2026. doi:10.1001/jamahealthforum.2026.0420

Data
Data available: Yes
Data types: Data (not involving human participants)
How to access data: Supplement eTable 1 includes the state statutes, administrative regulations, and reimbursement policies for each state
When available: With publication

Supporting Documents
Document types: None

Additional Information
Who can access the data: underlying study data can be provided as an electronic supplement
Types of analyses: systematic legal mapping analysis
Mechanisms of data availability: included in supplemental materials
Any additional restrictions: None
